# Can Ethograms Be Automatically Generated Using Body Acceleration Data from Free-Ranging Birds?

**DOI:** 10.1371/journal.pone.0005379

**Published:** 2009-04-30

**Authors:** Kentaro Q. Sakamoto, Katsufumi Sato, Mayumi Ishizuka, Yutaka Watanuki, Akinori Takahashi, Francis Daunt, Sarah Wanless

**Affiliations:** 1 Graduate School of Veterinary Medicine, Hokkaido University, Sapporo, Hokkaido, Japan; 2 International Coastal Research Center, Ocean Research Institute, The University of Tokyo, Otsuchi, Iwate, Japan; 3 Graduate School of Fisheries Science, Hokkaido University, Hakodate, Hokkaido, Japan; 4 National Institute of Polar Research, Itabashi, Tokyo, Japan; 5 Centre for Ecology and Hydrology, Bush Estate, Penicuik, Midlothian, United Kingdom; University of Rennes 1, France

## Abstract

An ethogram is a catalogue of discrete behaviors typically employed by a species. Traditionally animal behavior has been recorded by observing study individuals directly. However, this approach is difficult, often impossible, in the case of behaviors which occur in remote areas and/or at great depth or altitude. The recent development of increasingly sophisticated, animal-borne data loggers, has started to overcome this problem. Accelerometers are particularly useful in this respect because they can record the dynamic motion of a body in e.g. flight, walking, or swimming. However, classifying behavior using body acceleration characteristics typically requires prior knowledge of the behavior of free-ranging animals. Here, we demonstrate an automated procedure to categorize behavior from body acceleration, together with the release of a user-friendly computer application, “Ethographer”. We evaluated its performance using longitudinal acceleration data collected from a foot-propelled diving seabird, the European shag, *Phalacrocorax aristotelis*. The time series data were converted into a spectrum by continuous wavelet transformation. Then, each second of the spectrum was categorized into one of 20 behavior groups by unsupervised cluster analysis, using *k*-means methods. The typical behaviors extracted were characterized by the periodicities of body acceleration. Each categorized behavior was assumed to correspond to when the bird was on land, in flight, on the sea surface, diving and so on. The behaviors classified by the procedures accorded well with those independently defined from depth profiles. Because our approach is performed by unsupervised computation of the data, it has the potential to detect previously unknown types of behavior and unknown sequences of some behaviors.

## Introduction

Assessing animal behavior is essential to understand animal life. The initial process of studying behavior is to make a catalogue of the discrete behaviors typically employed by a species, namely an ethogram. Making an ethogram by direct observation is fundamental to understanding animal behavior [1]. However, this approach is not feasible for some flying or diving animals that often spend considerable portions of their lives beyond the limit of human vision. In such cases, indirect observation via biotelemetry allows researchers to monitor animal behavior remotely [2]. Furthermore, the development of animal-borne data loggers allows vast amounts of behavioral and physical information to be recorded on multiple sensors as an animal moves through its environment. (i.e., biologging) [3]–[5]. Several types of devices, such as light sensors [6], digital cameras [7], and video cameras [8], have been attached to animals to monitor precise behaviors. One of the most powerful devices used to monitor free-ranging behavior is the animal-borne accelerometer, which records both static and dynamic acceleration. Static acceleration is derived from an animal's body pitch, while dynamic acceleration is derived from body movement. From these two parameters, researchers can readily identify a range of discrete behaviors [9]–[13].

To our knowledge, the first study to recognize the potential of recording acceleration to determine behavior patterns was that of Yoda et al. [9], in which bi-axial accelerometers were used to differentiate whether Adélie penguins *Pygoscelis adeliae* were upright, prone, walking, porpoising, or tobogganing. The authors discriminated each behavior by visual observation of acceleration data. A more sophisticated technique was presented by Watanabe et al. [11] for the domestic cat *Felis catus*. They employed fast Fourier transformation to characterize acceleration signals and discriminated several behavior patterns by a stepwise canonical discriminant analysis supported by video-recorded data. More recently, a simple method was proposed to identify behaviors from a decision tree based on an acceleration signal feature [12], [13]. All these procedures require the researcher to first create the criteria to identify each behavior [11]–[14], and when a full set of information about the behavior is available, this approach is very effective. However, it is often difficult to obtain adequate amounts of information to determine behavioral criteria for animals that spend much of their time out of sight of a human observer, for example in the open ocean.

To overcome this shortcoming, we adopted a novel two-stage approach to identifying animal behavior with accelerometers. Acceleration signals were first categorized into several behavioral groups by an unsupervised classification algorithm, and then these groups were interpreted by the researcher for example as walking, swimming, sleeping etc. (see Guilford et al. [15]). However, in some cases behaviors cannot readily be interpreted because information on basic behavior patterns is lacking. For instance, humans have never directly observed sperm whale *Physeter macrocephalus* behavior at 1000 m depth. Therefore, researchers can interpret sperm whale behavior only indirectly using clues obtained. These are called putative behaviors. However, even if it is currently impossible to directly observe a certain type of behavior, categorizing it as distinct from other familiar behaviors is an important first step to building a picture of how the animal functions and interacts with its environment.

The oscillation of acceleration signals derived from animal movement usually changes in periodicity and intensity. The process we adopted was to generate a spectrum from acceleration signals based on the continuous wavelet transformation, and then group each second of the spectrum by an unsupervised classification algorithm, the *k*-means clustering method. For oscillation analysis, the fast Fourier transformation has been widely employed (e.g. sound analysis) [11]. However, compared to sound data, body acceleration data are characterized by far fewer oscillations for each unit of time (i.e., for each distinct behavior). Therefore to precisely assess behavior at a localized time scale, continuous wavelet transformation is preferable as it allows small changes to be detected over short time frames. Continuous wavelet transformation is similar to fast Fourier transformation; the major difference is that Fourier transformations are assumed to apply to a static oscillation signals, whereas continuous wavelet transformations are not. Although a window function enables the Fourier transformation to localize an estimation of spectra in time, the number of oscillations in the signal used for the analysis is dependent on the signal frequency within the window. Therefore, the sensitivity for detecting an oscillation is not same among different frequency signals within the window. By comparing continuous wavelet transformations of fluctuating oscillations with Fourier transformation, it has been shown that the wavelet spectrum provides an unbiased and consistent estimation of the Fourier spectrum [16]. Recently, continuous wavelet transformation has been applied to oscillation analysis of climate change [17], disease vectors [18], and population trends [19], [20]. *K*-means clustering is a well known method that is efficient for large data sets and which can be performed in an unsupervised way with the number of clusters as a parameter [21].

To examine the validity of our procedures, we collected acceleration data from a medium-sized, foot-propelled diving seabird, the European shag *Phalacrocorax aristotelis*. Body acceleration signals associated with different behaviors have previously been classified [22], [23], making this species an ideal subject for the study. In this paper, we demonstrate our newly developed procedure for generating ethograms from body acceleration data and evaluate its performance through a comparison with the known behavior profile of the European shag.

Japanese translation of this paper is available in Text S1.

## Methods

### Ethics statement

This study was carried out under Research Permits 15/R/38 and MON/RP/69 and Bird Scientific Licences 4480 and 6676 issued by Scottish Natural Heritage.

### The study species

The foraging activity of European shags at the breeding colony on the Isle of May off the south-east coast of Scotland, UK, (56°11′N, 02°33′W), has been studied extensively using VHF telemetry and a variety of animal-borne logging devices including accelerometers [22]–[26]. Birds feed diurnally, typically making 1–4 foraging trips per day during chick rearing [24], and food for the brood is transported back to the colony in the parent's stomach [25]–[28]. They are foot-propelled pursuit-divers and, at this colony, mainly feed benthically on small fish such as the lesser sandeel (*Ammodytes marinus*) and butterfish (*Pholis gunnellus*). Whilst diving, descent and ascent through the water column is almost vertical. The frequency of the foot stroke used for propulsion decreases significantly with depth from 5 to 2 Hz (0.2–0.5 sec cycle) [22]. Shags typically fly to and from the foraging area using a continuous wing stroke of ca. 5.5 Hz (0.18 sec cycle) [23].

### Field work

Field work was conducted on the Isle of May, Scotland during the 2003 and 2006 breeding seasons. Birds (14 in 2003 and two in 2006) brooding small to medium-sized chicks were captured on the nest using a crook on the end of a long pole. Birds were sexed from voice and size (male vocalize and are larger than females), and a data logger was attached to the central back feathers by fixing two plastic cable ties and waterproof tape to a piece of plastic netting (3 cm×5 cm) glued to the feathers with a fast-setting glue in 2003 and waterproof tape only in 2006. Handling time was less than 10 min, and after release every bird returned to its nest immediately before voluntarily departing on one or more foraging trips during daylight hours. Instrumented birds were recaptured in the evening after completion of their final trip of the day, or the following day, and the loggers were retrieved.

### Instruments

Acceleration data loggers (M190L-D2GT; Little Leonardo Ltd., Tokyo, Japan) were used to obtain detailed information on body acceleration over a 24 h period (maximum 28.2 h because of memory capacity). Each logger was 15 mm in diameter, 53 mm in length, had a mass of 18 g in air and recorded depth (1 Hz), two-dimensional acceleration (64 Hz, only one axis was used in this study), and temperature (data not used in this study). It is possible that energy expenditure during a trip might be increased in instrumented birds. However, the mass of the data logger in air was only ca. 1% of the body mass of male and female European shags (1896±121 and 1619±99 g respectively; means±s.d.), and weighed markedly less than the typical load of fish brought back after a foraging trip (mean 106 g) [27]. Furthermore, there was no evidence of any obvious disruption to attendance behavior in the instrumented birds. We therefore assumed that the data collected were representative of normal behavior.

**Figure 1:**
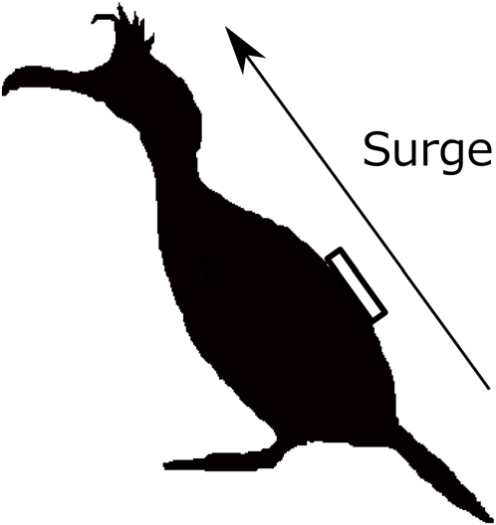
Position of the logger attachment and direction of the longitudinal (surge) body axis.

**Figure 2:**
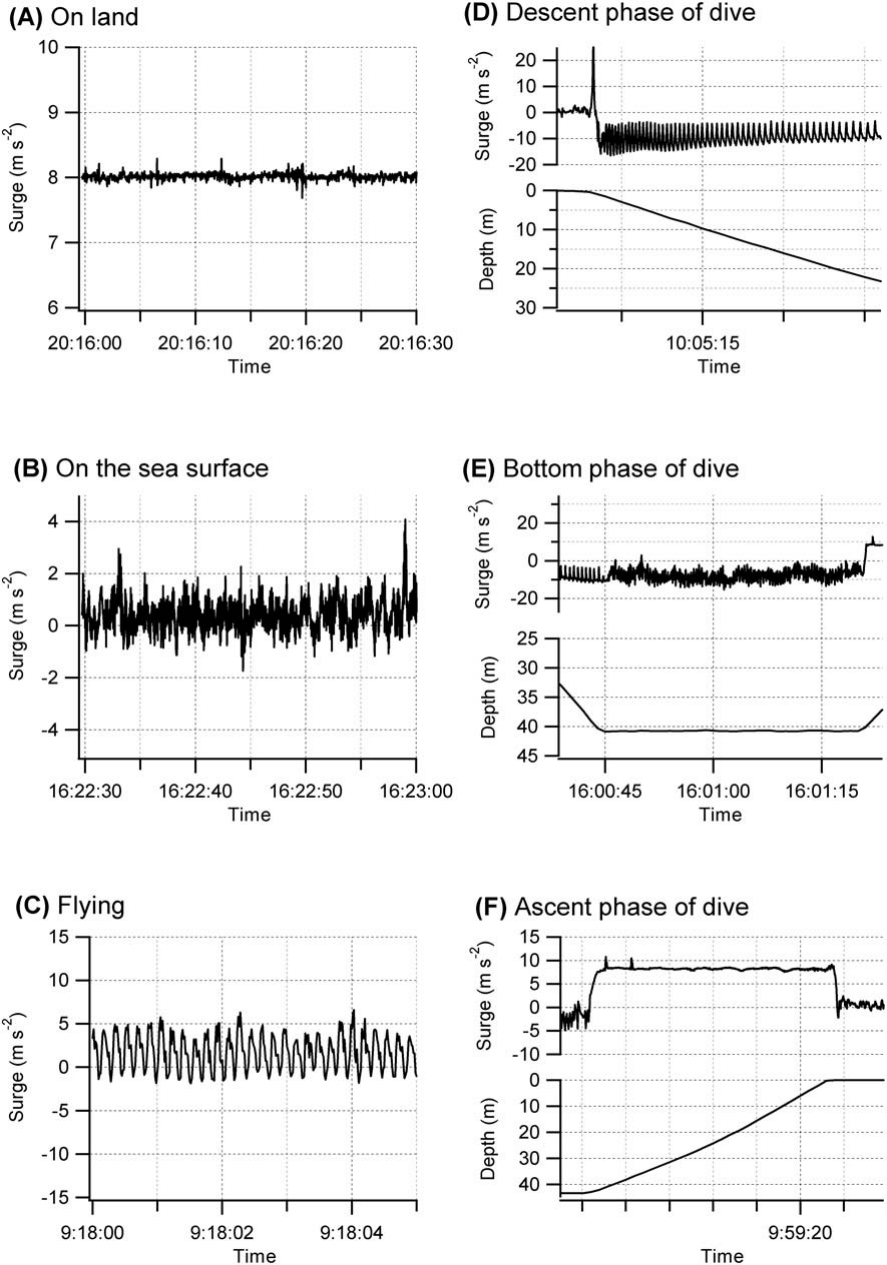
Surge and depth profiles of European shags. Figures represent behavior (A) on land, (B) on the sea surface, (C) in flight, (D) descending in a dive, (E) in the bottom phase of a dive, and (F) ascending from a dive.

Loggers were positioned so as to detect longitudinal (surge) accelerations (Fig. 1). Values recorded by the accelerometers were converted into acceleration (m s^−2^) with linear regression equations. To obtain the calibration equations, values recorded by each logger at 90° and −90° from the horizontal were regressed against the corresponding acceleration (9.8 m s^−2^ and −9.8 m s^−2^, respectively). Loggers measured both dynamic acceleration (such as wing stroking activity) and static acceleration (such as gravity). Thus, the amplitude of the surge acceleration represents the component of gravitational acceleration that changes in response to the posture of the bird when not moving. This enabled us to determine the orientation of the tag, which in turn relates to the posture of a bird, i.e., whether it was standing up or sitting down. Several behavior patterns were identified by comparing the acceleration profiles by eye with known information (Fig. 2) [9], [22], [23]. The instrumented birds made between two and six foraging trips over their respective deployment periods. Dives were defined as bird movements to depths greater than 1 m.

### Continuous wavelet transformation

We applied continuous wavelet transformation to take into account the non-stationary oscillation of the acceleration data. The wavelet transform has been described “…as a ‘mathematical microscope’ in which one can observe different parts of the signal by just adjusting the focus” [29]. By decomposing a time-series into time and periodicity domains, the wavelet analysis can determine both the dominant modes of variability and how those modes vary in time [17]. A mother wavelet is a small wave that is well-defined in both time and periodicity and has a time-integral of zero. The continuous wavelet transform of a signal recorded as discrete sequence *x*_n_ is defined as the convolution of *x_n_* with a scaled and translated version of mother wavelet function *ψ*:
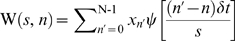
(1)where *s* is a scale, *n* is the localized time index, *δt* is the time step of a sequence, and *N* is the total number of data points [17]. By varying the wavelet scale *s* and translating along the localized time index *n*, a picture showing both the intensity of any feature versus the scale and how this intensity varies with time can be constructed.

**Figure 3:**
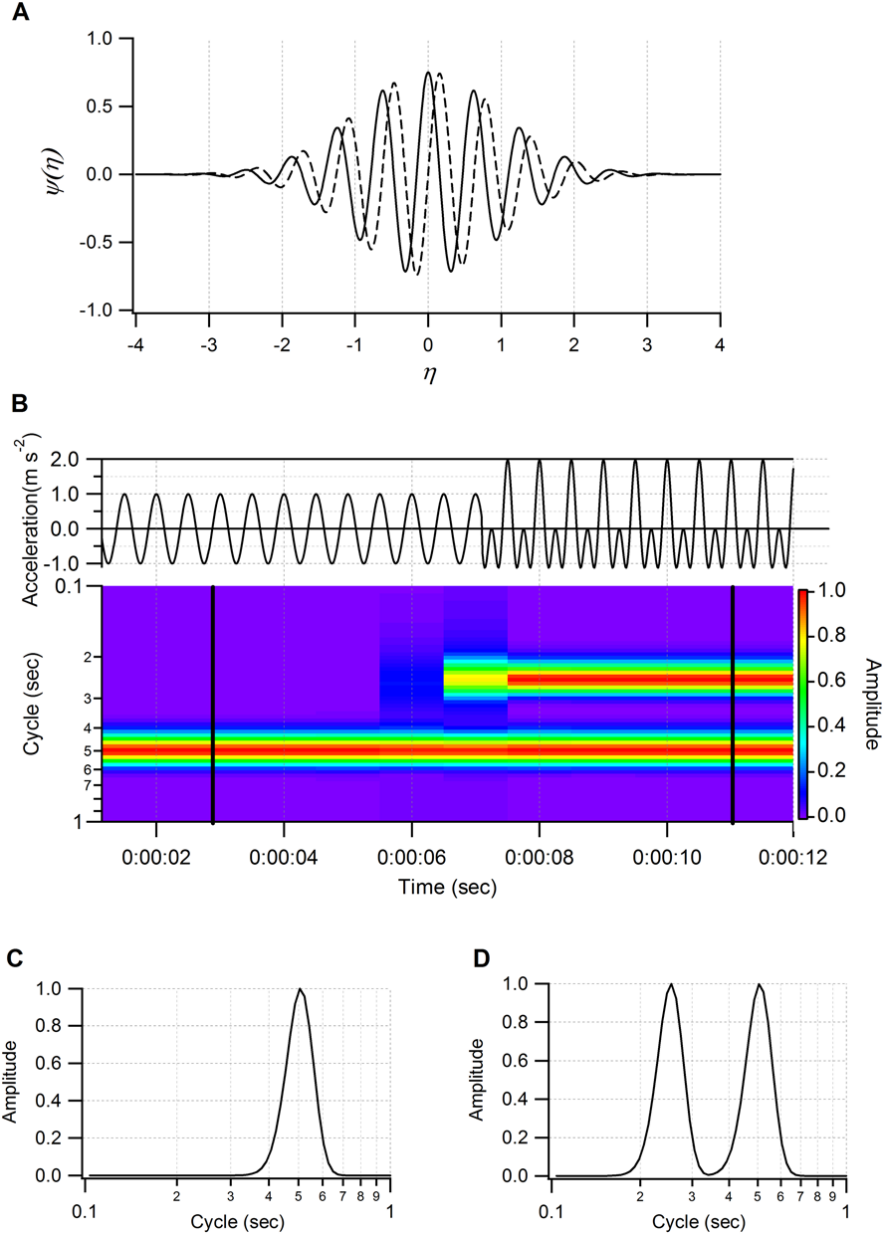
Schematic diagram of continuous wavelet transformation. (A) Morlet mother wavelet for ω_0_ = 10. Solid and dashed lines indicate real and imaginary parts respectively. (B) Example of behavior spectrum. Model data are constructed by a cosine function with a cycle of 0.5 sec (time< = 00:00:07) and two cosine functions with cycles of 0.25 and 0.5 sec (time>00:00:07) (top). Amplitudes of both cosine functions are 1. Behavior spectrum ranging from 0.1 to 1 sec was generated by model data (bottom). (C) Local behavior spectrum at 0:00:03 and (D) 0:00:11. The peak amplitude of the local behavior spectrum corresponds to the amplitude of original data at the corresponding cycle.

We analyzed temporal changes in the distribution of amplitude at a different scale *s* using the Morlet mother wavelet function. The Morlet wavelet function is:
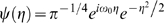
(2)where ω_0_ is the nondimensional frequency. The Morlet wavelet function is based on trigonometric principles (Fig. 3A). Therefore it can decompose time series data into several sine waves (Fig. 3B) and is widely used for the analysis of ecological processes [18]–[20]. The nondimentional frequency ω_0_ is the parameter which defines the balance of decomposition resolution between time and periodicity domains. The larger ω_0_ value has higher sensitivity in the periodicity domain and lower sensitivity in the time domain. The value ω_0_ = 10 was chosen to best differentiate the time and periodicity domains for the body acceleration of European shags. This mother wavelet consists of approximately seven waves (Fig. 3A). The signal was convoluted with this mother wavelet. Consequently, the sensitivity for both time and periodicity components was highest when the signal made up approximately seven waves.

To connect the wavelet coefficient W(*s*, *n*) to the amplitude of a signal, the formula was defined by:
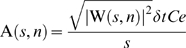
(3)where *Ce* is the empirical coefficient (1.06392). We added *Ce* into the formula to modify the value of A(*s*, *n*) equal to the amplitude of the oscillation in *x_n_* at the corresponding scale *s* and the localized time index *n* (Fig. 3B).

The Fourier wavelength (cycle) λ corresponding to the scale *s* is expressed as [17]:
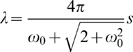
(4)Therefore, this formula can be transformed to:
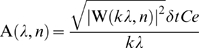
(5)and
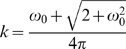
(6)


The value of A(λ, *n*) corresponds to the amplitude of the trigonometric function component in the signal with the cycle λ at the localized time index *n*. We calculated the average value of A(λ, *n*) within each sec by each cycle λ to reduce the data size and enable further clustering analysis, here termed a “behavior spectrum” (Fig. 3B, C, D). A behavior spectrum provides a measure of the contribution of each periodicity to the signal at a different time position by each sec. We calculated the values of a behavior spectrum at 64 time steps of a cycle ranging from 0.05 to 1 sec as:
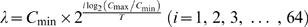
(7)where *T* is the number of time steps (64 steps), *C_min_* is the minimum cycle (0.05 sec), and *C_max_* is the maximum cycle (1 sec).

Each behavior was characterized by a behavior spectrum providing its periodicities and amplitude. The shape of a spectrum at each time point was determined by the features of the original signal. Usually, a signal derived from animal movement is not the shape of a single trigonometric function, but is made up of a number of sine waves of different amplitudes whose frequencies are integer multiples of each other. The lowest frequency is called the fundamental frequency and the higher frequencies are called harmonics (Fig. 3B). The signal at fundamental frequency was regarded as the cycle of body movement [30].

The amplitude calculated in this procedure is not directly related to the intensity of animal dynamic movement. However, the convolution with the Morlet wavelet function decomposes the acceleration signal into several sine waves and inversely the sum of decomposed sine waves becomes the same shape as the original acceleration within the range of cycles applied in the transformation. Therefore similar suits of sine waves in the decomposed data may indicate the same type of movement with slightly different periodicities and intensities [30]. By comparing the signal amplitudes among similar shapes of the behavior spectrum, the relative intensity of the movement could be assessed within the same type of movement.

The ‘cone of influence’ is a reflection of a consequent loss in the wavelet spectrum near the start and end of the time series [17]. To avoid the cone of influence, we truncated the behavior spectrum at the beginning and end region by 3 times the length of the maximum scale.

### Clustering

We employed the *k*-means algorithm to cluster the behavior spectra at each sec to generate the ethogram. *K*-means clustering is an unsupervised, interactive algorithm that minimizes the within-cluster sum of squared Euclidean distances from the cluster centroids. The algorithm is composed of the following steps [21]:

1. Place *k* points randomly in the space represented by the objects that are being clustered. These points represent initial group centroids.
2. Assign each object to the group that has the closest centroid.
3. When all objects have been assigned, recalculate the positions of the *k* centroids.
4. Repeat Steps 2 and 3 until the centroids no longer move. This produces a separation of the objects into groups from which the metric to be minimized can be calculated.

Behavior spectra at each time point were composed of cycles of 64 time steps. In other words, each spectrum was defined by 64 values. Therefore, it was mathematically the same as a point in 64 dimensions. We performed *k*-means clustering in the same manner as clustering points in 64 dimensions [31].

### Acceleration ethogram

To generate the acceleration ethogram, we used data from three male and three female European shags for which data were available for 24 h. Surge acceleration was converted to the behavior spectrum. We assumed 20 behavior elements would be adequate to cover all behavior patterns of European shags in the breeding season. The behavior spectra from the six birds were combined and processed by the *k*-means clustering algorithm to determine 20 centroids (clusters) in 64 dimensions. These centroids represented typical spectra of discriminated behaviors. We regarded these 20 spectra as an “acceleration ethogram”.

### Behavior discrimination by acceleration ethogram

Surge accelerations from sixteen birds were converted into a behavior spectrum. Each spectrum at each sec was regarded as a point in 64 dimensions, and calculated as squared Euclidean distances between the point and the 20 centroids that corresponded to the elements of the acceleration ethogram. The centroid providing the shortest squared Euclidean distance was chosen as the behavior element for the spectrum at each sec.

### Behavior discrimination by water depth

To evaluate the accuracy of our procedure we used independent information on time spent at depth by the sixteen birds, to construct detailed time budgets for each individual. A typical foraging trip for an European shag consists of a flight out from the breeding colony to the feeding site, followed by a series of dives with periods between dives spent on the sea surface, after which the bird returns to the colony [32]. Six behavior phases were defined from the water depth–time series: on land, commuting to and from the feeding area, dive descent, the bottom phase of the dive, dive ascent, and on the sea surface. Different phases within a dive were defined using the rate of change in depth (descent, less than −0.6 m s^−1^; bottom, −0.3 to +0.3 m s^−1^; ascent, >1 m s^−1^). Shags typically dive in bouts, and following Watanuki et al. [33], we used a bout-ending criterion of 340 secs to identify discrete bouts [34]. The interval between dives within a dive bout was defined as time spent on the sea surface. The durations of flights to and from a foraging site in a foraging trip were reported to be less than 30 min [32]. Therefore, 30 min before and after a dive bout were assumed to be the commuting phase. The remaining time was assumed to correspond to the period spent on land. The compositions of the behavior elements of the acceleration ethogram were calculated in each behavior phase defined by the depth profile.

### Computer application

To facilitate and perform the procedures in this study, we developed a user-friendly application called “Ethographer”. The code was written in Igor Pro language under Igor Pro ver. 5 (WaveMetrics Inc., Lake Oswego, OR, USA). The application works on an Igor Pro platform, provides a graphical user interface, and is easy to master. Ethographer is available, at no charge, for academic use (http://bre.soc.i.kyoto-u.ac.jp/bls/index.php?Ethographer).

## Results

**Figure 4:**
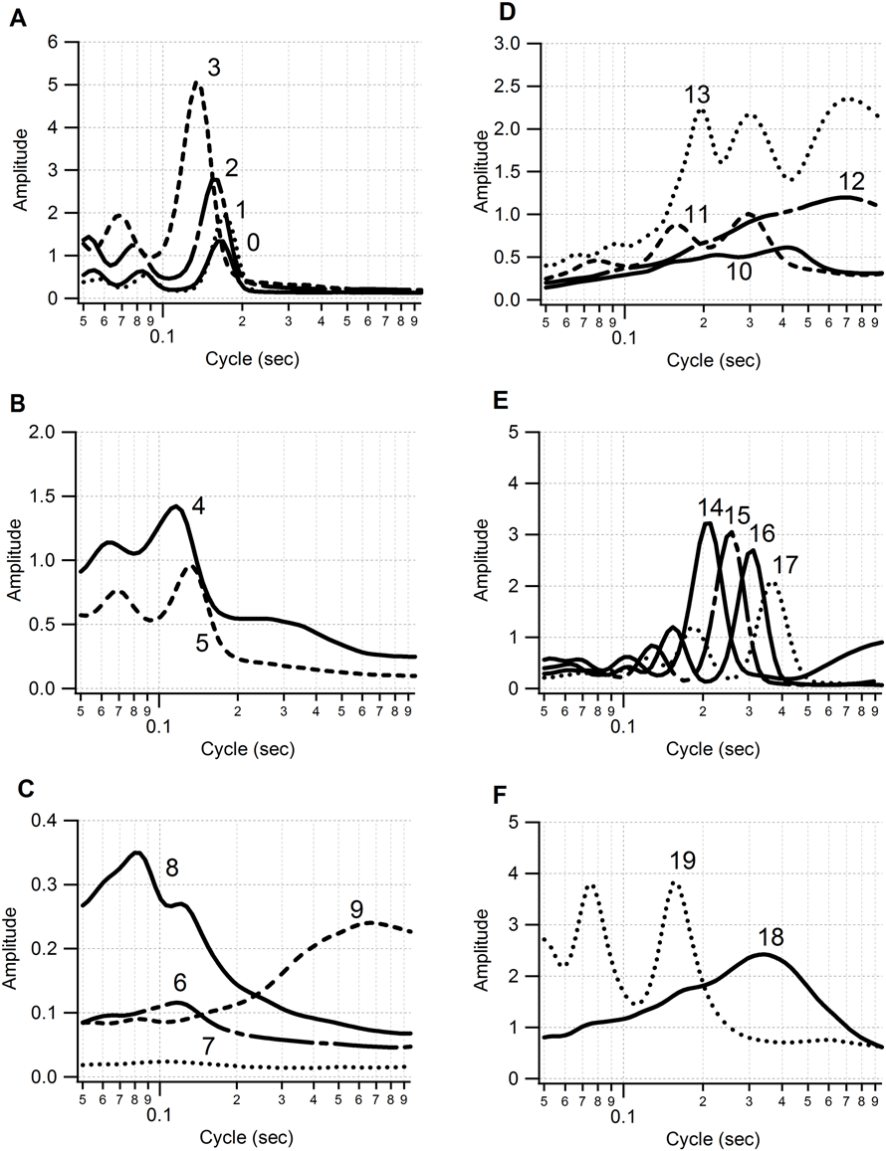
Twenty elements of acceleration ethogram. The ethogram was constructed from 24 h of surge acceleration data from six shags (3 males and 3 females). The vertical axis represents the amplitude of the acceleration. The horizontal axis represents the cycle length of the acceleration. The ethogram is separated into six figures for ease of visualization.

The surge acceleration signals were classified into 20 behavior groups in the acceleration ethogram. Each behavior element in an acceleration ethogram was described by the spectrum of an acceleration pattern (Fig. 4). The spectra were thought of as the ethogram derived from surge acceleration. The shapes of several elements in an acceleration ethogram seemed to be similar (e.g. elements 0, 1, 2; elements 14, 15, 16, 17) (Fig. 4). These elements may represent the same pattern of behavior with different periodicities and intensities of movement. The amplitude at the dominant cycle in an ethogram ranged from 0.024 (0.11 sec cycle in element 7) to 5.072 (0.13 sec cycle in element 3). In general, a behavior element producing a strong amplitude exhibited a clear peak, whereas a spectrum with a weak amplitude tended to be flatter in shape. This may mean that a discrete behavior with a strong dynamic movement is composed of periodic motions while a flat spectrum indicates that the original behavior consists of a less periodic movement.

**Figure 5:**
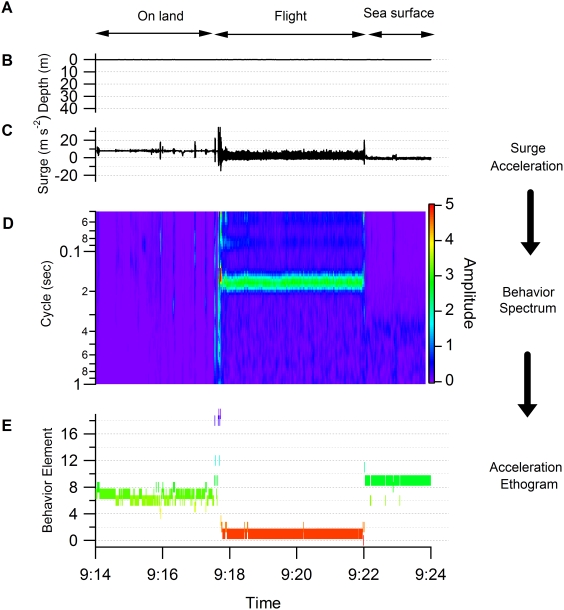
Flowchart of procedure to generate ethogram (data representing flight). (A) The behavior phases (on land, in flight and on the sea surface) were determined by data observation. (B) Depth. (C) Surge acceleration. (D) Behavior spectrum. (E) Behavior element as determined by acceleration ethogram. The elements were compared with the results of data observation.

**Figure 6:**
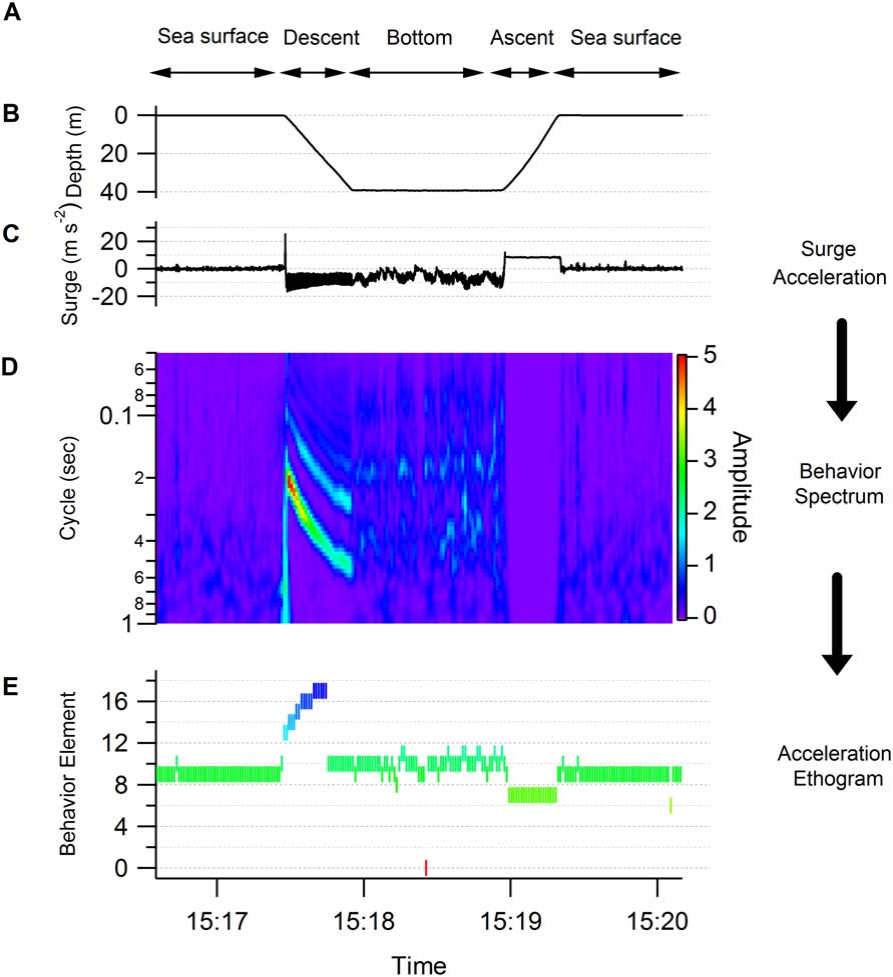
Flowchart of procedure to generate ethogram (data representing dive). (A) Behavior phases (on the sea surface, dive descent, bottom phase and dive ascent) were determined by data observation. (B) Depth. (C) Surge acceleration. (D) Behavior spectrum. (E) Behavior element as determined by acceleration ethogram. The elements were compared with the results of data observation.

**Figure 7:**
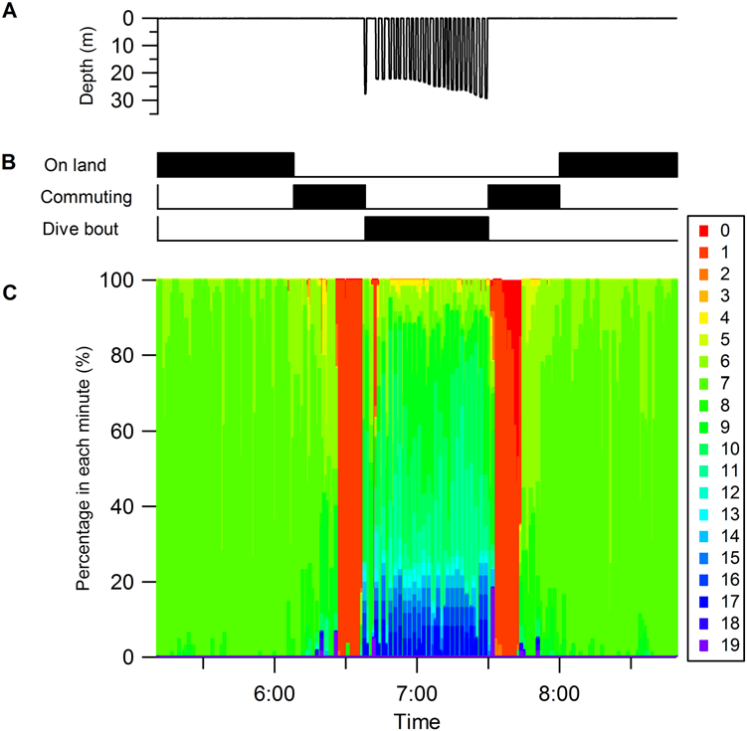
Correlation between acceleration ethogram and behavior definition by depth profile. (A) Depth. (B) Behavior phase defined by depth profile. The behavior phases of sea surface, descent, bottom and ascent were together defined as “Dive bout”. (C) Percentages of behavior elements in acceleration ethogram at each min.

Figure 5 shows an example of the application of our procedure. In the behavior spectrum, only a weak signal appears in the first section (Fig. 5D). This is equivalent to a weak signal in surge acceleration (Fig. 5C), indicating that the bird was motionless. In contrast, a clear peak is seen in the middle section with an approximately 0.18 sec cycle. This corresponds to the periodicity in the flight of the shag (5.5 Hz, [23]). In the last section, a weak and noisy signal is seen in the spectrum, indicating the existence of non- or weakly-periodical movement with a cycle of more than 0.3 sec. This could be derived from the bird being in an unstable position, for example because it was on the sea surface. The lower panel shows the result of categorization by the *k*-means clustering algorithm (Fig. 5E). Behaviors were clearly divided into three parts as shown in the left, middle and right sections: time on land (elements 6 and 7), in flight (element 1), and on the sea surface (element 9). Another example is shown in Fig. 6 in which the behavior spectra were apparently discriminated into four parts: on the sea surface, dive descent, bottom phase, and dive ascent (Fig. 6D). In the descent phase, the dominant cycle changed in relation to the water depth from a 0.2 sec cycle at the surface to a 0.5 sec cycle at 40 m. These data were consistent with a shag descending using foot propulsion at 2–5 Hz (0.2–0.5 sec cycle), such that the frequency decreased with depth [22]. The spectrum in the bottom phase was characterized by a noisy signal with obscure peaks in 0.4 and 0.2 sec cycles. There was no peak in the spectrum of the ascent phase. Three behaviors (on the sea surface, bottom phase, and dive ascent) were well categorized by *k*-means clustering: element 9, mainly element 10, and element 7 respectively. Of particular interest in the *k*-means clustering were the results for dive descent where behavior spectra were categorized into several elements sequentially (elements 13, 14, 15, 16, 17, and 10). Among the behavior spectra of these elements, element 13 was characterized by several peaks and large amplitude (Fig. 4D). Spectrum shapes were similar among elements 14–17. However, the dominant cycles became longer and the amplitudes became smaller from element 14 to element 17. The dominant cycle of element 10 was the longest with the smallest amplitude. The sequence of these elements was consistent with known foot-propulsion behavior during descent, which changes in periodicity and intensity of movement with depth [22]. When visualized over the whole foraging trip, elements 0, 1, and 2, which produced an approximately 1.8 sec cycle, were exhibited before and after a dive bout, indicating continuous flight (Fig. 7). Elements 14, 15, 16, and 17 were associated with dive bouts and may have indicated dive descent.

**Figure 8:**
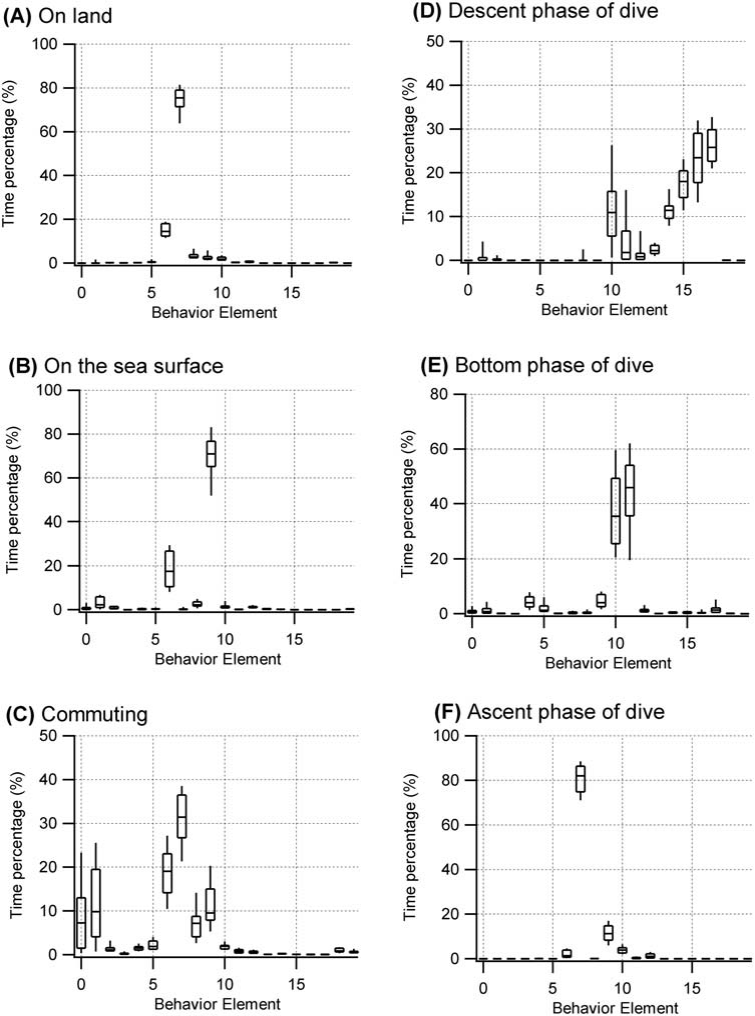
Compositions of behavior elements in different behavior phases. Box plots for compositions of the behavior elements of the acceleration ethogram in different behavior phases (n = 16). Each behavior phase was defined by a depth profile. Figures represent the compositions of the elements in the behavior of (A) on land, (B) on the sea surface, (C) commuting between colony and foraging site, (D) descending in a dive, (E) bottom phase of a dive, and (F) ascending from a dive. The box plot shows the median (center line), the upper and lower quartiles (edges of the box), and 10% and 90% percentiles (ends of whiskers).

Behavior phases defined by the depth profile exhibited different compositions of behavior elements in the acceleration ethogram (Fig. 8). Several behavior phases were composed of one or two behavior elements: on land (elements 6 and 7), on the sea surface (elements 6 and 9), bottom phase (elements 10 and 11), and ascent (element 7). Interestingly, the relative proportions of elements 10 and 11 in the bottom phase of the dive varied considerably among individual birds (Fig. 8). Element 10 was linked to less periodic behavior and element 11 represented 0.3 sec cycle motion (Fig. 4). European shags on the Isle of May are known to be benthic feeders and to use two distinct foraging habitats in sandy and rocky areas [33]. Their foraging behavior differs markedly between these habitats such that in rocky areas birds typically travel horizontally over the seabed while in sandy areas they focus on a particular spot during the bottom phase. Currently we do not have sufficient information to identify the exact behaviors indicated by element 10 and 11 but individual differences in foraging habitat usage could potentially explain the high level of individual variation. The descent phase contained several elements, which corresponded to a change in the foot-propulsion cycle and intensity (Fig. 6 and Fig. 8D). The commuting phase (Fig. 8C) contained the most elements 0, 1, 6, 7, 8, and 9. This phase was defined as the time before and after a dive bout, and had a fixed duration of 30 min, and could thus contain not only flight, but also time spent on land and on the sea surface. Components of element 7 and element 9 in the commuting phase are likely to derive from land-based and sea surface behavior respectively (see Fig. 7). The unique elements in the commuting phase were elements 0 and 1. The dominant cycle of these elements was a 0.18 sec cycle, which is consistent with the dominant cycle of flight in European shags.

It is worth noting that acceleration signals when birds are on the sea surface could potentially vary due to environmental conditions such as sea state. Although we did not find such variability in our data set, such differences might be recognized as different behavior spectrum and thus ‘sea surface behavior’ could be categorized into more than one acceleration ethogram.

## Discussion

When our procedure is adopted to generate an ethogram, four parameters need adjusting to take account of the characteristics of the species under consideration. The first is the cycle range of body acceleration for continuous wavelet transformation. We selected a cycle range from 0.05 to 1 second for European shags. This range corresponds mainly to dynamic body acceleration in this species [22]. The second parameter is the number of elements generated by the *k*-means clustering algorithm. The shapes of major spectra in an acceleration ethogram obtained as the output of *k*-means clustering became stable by increasing the number of behavior elements to 20, but as a consequence, two similar shaped spectra were generated. Therefore we set 20 as the number of behavior element to cover all behavior patterns. Because *k*-means clustering is processed by mathematical definition, the clustering result sometimes seems to be different from what would be expected. The practical solution would be to set a larger number of elements to apply to the *k*-means algorithm than is strictly necessary, and then to combine the elements that the researcher identifies that represent the same behavior. The third parameter is the ω_0_ value in the Morlet mother wavelet function. Owing to the process of continuous wavelet transformation, detection of periodical movement was preferable to non-periodical movement. Adjustment of the ω_0_ parameter would change the sensitivity to time and periodicity in the movements. The fourth parameter is the time duration of a behavior spectrum. We calculated the behavior spectrum by averaging the value of A(λ, *n*) within each sec, assuming enough fine scale sensitivity to describe each discrete behavior of the European shag for general purposes. The time duration for averaging should be adjusted by taking the minimum duration of each discrete behavior in the species into consideration. The computer application, Ethographer, allows these parameter values to be changed.

The *k*-means algorithm assumes the same periodicity with the same amplitude within a behavior element. Therefore, for instance, it is difficult to discriminate precisely between walking and running because behavior changes gradually along a continuum. The algorithm will generate several elements with slightly different periodicities for walking or running (see the descent section in Fig. 6D). In this case, the exact definitions among elements may vary between analyses.

We were aware of a few constraints caused by computer resources, the most important of which was the amount of data that can be applied to a *k*-means clustering algorithm. In our analysis we used 24-hour blocks of data from six out of 16 shags to apply to the algorithm. The amount of data used to generate the acceleration ethogram from this subsample was ca. 500,000 time points, which was the maximum amount our computer (Intel Core 2 Duo 6600 CPU, 2 GB RAM) was able to process. Another constraint relates to the *k*-means clustering algorithm where the output of clustering is not always exactly the same, although the outputs estimated from the same data set in different analysis sessions are similar. The reason is that *k*-means clustering does not estimate the optimized solution, but rather the approximate optimized solution. In terms of the amount of computation, we considered that estimation of the optimized solution was not practical (e.g. hierarchical clustering).

The categorization of behavior from body acceleration coincided well with the behavior definition based on the water depth–time series. However, it was impossible to discriminate between time spent on land and dive ascent in our approach. We used mainly the dynamic acceleration component of the surge to produce the behavior spectrum. Therefore, when an animal was motionless, the spectrum was the same even if its posture differed. The acceleration ethogram produced in this study was the behavior catalogue derived solely using data for surge acceleration of the bird's body. To make a complete ethogram, posture information obtained from the long periodicity component of body acceleration, as well as use of a time-depth recorders and GPS loggers, would be required.

To date, quantifying the behavior of wild animals that are hard to track has been extremely challenging. In the context of conservation biology, lack of information about the foraging ecology of an endangered species may hinder the development of an effective conservation strategy. Our approach has the potential to shed light on hitherto unknown aspects of the lives of such animals. It is noteworthy that our procedure employs an unsupervised clustering algorithm opening up the possibility to extract novel behavior patterns that researchers have never observed directly. Although the present system does not guarantee the successful generation of an ethogram for other species, our approach offers considerable potential to study the behavior of poorly known species, especially in the case of the animals living far from human observation.

## Supporting Information

Text S1 Japanese translation of this article.
(0.48 MB PDF)
