# Supplementary material for: Can Ethograms Be Automatically Generated Using Body Acceleration Data from Free-Ranging Birds?
Source: PLoS One. 2009 Apr 30;4(4):e5379. doi: 10.1371/journal.pone.0005379 (PMC2671159; doi:10.1371/journal.pone.0005379)
Supplement: Text S1 — Japanese translation of this article. (0.48 MB PDF) [file pone.0005379.s001.pdf]

Title

Can Ethograms Be Automatically Generated Using Body Acceleration Data from Free-Ranging Birds?

（和訳）

体の加速度情報から野外で行動する鳥類のエソグラムを自動作成する試み

Authors and Affiliations

Kentaro Q Sakamoto[1]*, Katsufumi Sato[2], Mayumi Ishizuka[1], Yutaka Watanuki[3], Akinori Takahashi[4], Francis Daunt[5], Sarah Wanless[5]

(1) Graduate School of Veterinary Medicine, Hokkaido University, Sapporo, Hokkaido, Japan

(2) International Coastal Research Center, Ocean Research Institute, The University of Tokyo, Otsuchi, Iwate, Japan

(3) Graduate School of Fisheries Science, Hokkaido University, Hakodate, Hokkaido, Japan.

(4) National Institute of Polar Research, Itabashi, Tokyo, Japan.

(5) Centre for Ecology and Hydrology, Bush Estate, Penicuik, Midlothian, United Kingdom.

* Corresponding author: Kentaro Q Sakamoto

E-mail: sakamoto@vetmed.hokudai.ac.jp

Abstract

エソグラムとは、個々の動物種に特有で典型的な行動をまとめたカタログである。動物行動の記録は、伝統的に対象種を直接観察することによって行われてきた。ただし、深海や空中など人間の視界から遠く離れた場所で為される行動の場合、直接観察による行動の記録は難しく、不可能な場合も多い。動物装着型データロガーが近年洗練されてきたことによって、この問題は克服されつつある。特に、加速度計は飛翔・歩行・遊泳などに伴う体の動的加速度を記録することが可能なため、行動を記録するのに役に立つ。しかし、これまでは対象動物の野外における行動がどのようなものであるかが事前に分かっていなければ、体の加速度データを元に行動を分類することは出来なかった。私達は、本論文で体の加速度を元に行動を自動分類する方法を示すと共に、この方法を簡単に実行することが出来る Ethographer というコンピューターソフトウェアを公開した。足漕ぎによって潜水を行う海鳥のヨーロッパヒメウ（*Phalacrocorax aristotelis*）の体の長軸方向の加速度を用いて、本方法の性能を評価した。まず、得られた時系列加速度データを連続ウェーブレット変換によ

ってスペクトルに変換した。その後、1 秒ごとに計算したスペクトルを教師なし分類法（*k*-means 法）によって 20 個のグループに分類した。典型的なものとして抽出された行動は、体の加速度の周期によって記述することができた。それぞれの行動は、対象個体が陸上での休息、飛行、海面での休息、潜水などを行っている行動に対応していると考えられた。本方法で分類された行動は、深度データを元に独立に定義した行動とよく一致した。本方法は教師なし分類法を用いていることから、これまで知られていなかった行動や未知の行動連鎖を検出できる可能性がある。

Introduction

動物の生活を理解するには、その行動を調べることが必要不可欠である。行動を研究する上で、まず最初に行うことは、対象種によって為される特有の行動を分類し、カタログを作成することである。このカタログをエソグラムという。対象を直接観察しエソグラムを作成することは、その行動を理解するための根本的な作業である[1]。しかし、人間が観察できないところで生活の大部分を営む飛翔動物や潜水動物の場合、この方法は現実的ではない。そのような場合は、バイオテレメトリーを用いた間接的な観察によって、遠隔から動物の行動を調べることが可能である[2]。加えて、近年、動物装着型データロガー（センサー付き記録装置）を用いることによって、動物が移動する間に計測される

行動・環境情報を記録することができるようになった（これをバイオロギングと言う[3-5]）。データロガーが発達し、多様なセンサーを用いることによって大量の行動学的、生理学的情報を計測・記録することが可能となってきた。精密に行動を記録するために、照度計[6]、デジタルカメラ[7]、ビデオカメラ[8]などが搭載されたデータロガーが、これまで動物に装着されてきた。これらの装置の中で、動物の行動を野外で記録するのに最も力を発揮する装置の 1 つとして加速度計がある。加速度計は、静的加速度（static acceleration）と動的加速度（dynamic acceleration）の両方を同時に記録する。静的加速度は動物の体軸角度に由来し、動的加速度は体の動きに由来する。この二つの情報から、さまざまな行動パターンを判別することが出来る[9-13]。

私達が知る限り、複数の行動パターンの判別を目的として加速度計を用いた最初の試みは、Yoda et al. [9]によるものである。彼らの研究では、2 軸記録型の加速度計を用いて、アデリーペンギン（*Pygoscelis adeliae*）の行動（直立姿勢、腹這い姿勢、歩行、イルカ泳ぎ、トボガン歩行）を判別した。この研究で、著者らは記録された加速度データを眼で確認することによって、それぞれの行動パターンを判別した。その後、イエネコ（*Felis catus*）を対象として、さらに洗練された方法が Watanabe et al. [11]によって発表された。彼らは事前に加速度計が装着された動物の行動をビデオカメラで撮影して、それぞれの行動パターンに対応する加速度信号を得た。その後、高速フーリエ変換を用いて加速度信号が持つ周波数特性を抽出し、抽出された情報に対してステップワイズ正準判別分析を行うことで、数種類の行動パターンを区別した。さらに最近では、加

速度信号の特徴から決定樹を作成し、これを元に行動を判別する簡便な方法も考案された[12,13]。これら全ての方法は、行動の判別に際して、研究者が事前に判別基準を作成する必要があった[11-14]。動物が取りうる行動について充分な情報を手に入れることができる場合であれば、これらの方法は非常に有用であろう。しかしながら、海洋など、観察者の視界から遠く離れた場所で多くの時間を過ごす動物の場合、行動判別の基準を作成するために充分な情報を得ることは、しばしば困難である。

この欠点を克服するために、私達は加速度計から動物の行動を判別するための新しい方法を考案した。この方法は二つの手順より構成される。まず、最初の工程で加速度信号を教師なし分類法により複数の行動グループに分類する。その後、分類されたグループがいかなる行動を意味するのか、研究者が解釈する（例えば、歩行や遊泳、睡眠など）（Guilford et al. [15]を見よ）。基本的な行動パターンについての知見が欠けている場合、分類された行動パターンが何を意味するか直ちには解釈できない場合もある。たとえば、深度 1,000m におけるマッコウクジラ（*Physeter macrocephalus*）の行動を直接観察した人間はいない。したがって、研究者は得られた手がかりだけを頼りにマッコウクジラの行動を解釈しているのである。このような行動を putative behavior と呼ぶ。しかし、たとえ特定の種類の行動パターンが今の段階では直接観察できないとしても、その行動パターンを他の行動パターンとは明瞭に違うものとして区別し、分類することは、その動物がどのように生き、また周辺環境と関係し合っているかを描き出すための重要な最初の一歩となるはずである。

動物の動きに由来する加速度信号における振動は通常、周期と強さが変動する。私達が考案した方法では、加速度信号を連続ウェーブレット変換（continuous wavelet transformation）して 1 秒ごとのスペクトルを計算し､その後、スペクトルを教師なし分類法（*k*-means 法）によって分類した。振動データの解析では、高速フーリエ変換が広く用いられている（例えば、音声の解析）[11]。しかし、音声情報に比べると、体の加速度信号は単位時間（例えば、それぞれの行動パターン）当たりで非常にわずかな振動しか有していない。従って、それぞれの時刻における行動を高精度に感知するためには、短時間に発生した小さい変化を検出することの出来る連続ウェーブレット変換の方が好ましい。連続ウェーブレット変換は高速フーリエ変換に類似の方法であるが、フーリエ変換は定常的な振動に適用されることを想定しているのに対して、連続ウェーブレット変換はそうでない、ということが大きな違いとして挙げられる。フーリエ変換でも窓関数を用いることで、それぞれの時刻における局所的なスペクトルを算出することができるが、変換に用いる振動の数は窓関数内における信号の周波数に依存する。従って、振動を検出する感度は、窓関数内における信号の周波数が異なれば変化してしまう。振動の性状が変動する信号のスペクトルを計算する場合、フーリエ変換に比べて連続ウェーブレット変換では、バイアスが少なく、安定した状態でスペクトルを計算できるといえる[16]。最近、気候変動[17]や伝染病伝播[18]、個体群動態[19,20]の分野で振動現象を解析するために、連続ウェーブレット変換は用いられるようになってきた。*k*-means 法は、大

規模データを解析するのに適した方法としてよく知られており、生成するグループ数を変数として設定することで教師なし分類を行うことができる[21]。

私達の方法の有効性を試験するために、ヨーロッパヒメウ（*Phalacrocorax aristotelis*）の体の加速度データを計測した。ヨーロッパヒメウは、足漕ぎによって潜水を行う中型の海鳥である。個々の行動パターンによって生み出される体の加速度の特徴は過去に明らかになっているため[22,23]、この種は本研究に理想的な動物種であった。本論文では、体の加速度よりエソグラムを生成する私達の新しい方法を示すと共に、ヨーロッパヒメウの既に知られた行動パターンとの比較を通じて、その性能を評価する。

Methods

Ethics statement

本研究はScottish Natural Heritage によって発行されたResearch Permits 15/R/38とMON/RP/69及びBird Scientific Licences 4480と6676の許可の元に遂行された。

The study species

野外調査地（英国スコットランドの南東部沖にあるメイ島、56°11' N, 02°33' W）におけるヨーロッパヒメウの採餌活動はVHFテレメトリーおよび加速度計を含む多種の動物装着型データロガーによって広く研究が行われて来た

[22-26]。対象種は、日中に採餌を行う。育雛期間中は、1日当たりおよそ1~4回の採餌トリップを行う[24]。採餌後、ヒナへ給餌するための餌を胃に一時的に蓄え、営巣地へ運ぶ[25-28]。彼らは足漕ぎによって潜水を行い、この営巣地ではlesser sandeel (*Ammodytes marinus*)やbutterfish (*Pholis gunnellus*)といった海底に生息する小型魚類を主に餌としている。水中で潜行と浮上を行う時には、水中をほぼ垂直に移動する。水中での推進のための足漕ぎ周波数は、深度と共に5Hzから2Hz（0.2から0.5秒周期）へと著しく減少する[22]。また、ヒメウは採餌場所へは飛行により移動し、この時には約5.5Hz（0.18秒周期）の連続的な羽ばたきを行う[23]。

Field work

野外調査は、2003年と2006年の繁殖期に、英国スコットランドのメイ島で行った。小型から中型のヒナを育雛している個体を研究に用いた（2003年に14羽と2006年に2羽）。長い棒の先端に金属製の鉤を装着した器具を用いて、巣にいる個体を捕獲した。鳴き声と体サイズから性判定を行った（オスは鳴き、また体がメスに比べて大きい）。2003年には、データロガーを2本のプラスチッククケーブルタイと防水テープを用いてプラスチックネット（3cm x 5cm）に固定したものを、速乾性接着剤によって背中の中心部の羽毛に装着した。2006年には、防水テープのみを用いて装着した。ハンドリング時間は10分以内であった。放鳥後、全ての個体は直ちに巣にもどり、日中のうちに自発的に採餌トリップへ出かけた。データロガーを装着した個体は、その日の最後の採餌トリップが

終了した後、夜間もしくは翌日に再度捕獲し、データロガーを回収した。

Instruments

加速度データロガー（M190L-D2GT；リトルレオナルド、東京）を用いて、24時間以上の精密な体の加速度データを取得した（メモリ容量制限のため、最大28時間の記録）。データロガーは直径15mm、長径53mm、空気中での重量が18gで、深度（1Hz）、2軸の加速度（64Hz、本研究では1軸分のデータのみ用いた）、温度（1Hz、本研究ではデータを用いなかった）を記録するよう設定した。データロガーを装着したことによって、採餌トリップ中のエネルギー消費量が増大した可能性はあるが、空気中でのデータロガーの重量はヨーロッパヒメウの体重（オス1896 ± 121、メス1619 ± 99 g； 平均 ± 標準偏差）の約1%であり、採餌トリップの際にヒメウが持ち帰る魚の重量（平均106g）に比べて非常に軽かった[27]。その上、巣で観察された行動からは、データロガーの装着が対象個体に影響を及ぼしていることを示唆する知見は得られなかった。そのため、私達はデータロガーによって得られたデータは、ヨーロッパヒメウの正常な行動を反映していると想定した。

加速度計は、体の長軸方向（surge）の加速度成分を計測するように装着した（Fig. 1）。加速度計によって記録された値は、1次直線回帰式によって加速度（m s^{-2}）に変換した。変換式を得るため、水平方向に対して90度と-90度の角度になるように加速度計を置き、記録された値を得た。この時の加速度は、それぞれ、9.8 m s^{-2}と–9.8 m s^{-2}になるため、これを元に変換を行った。加速度計は、

動的加速度（羽ばたきなど）と静的加速度（重力など）の両方を計測する。従って、動きの無い状況であれば、surge方向の加速度は鳥の姿勢に応じた重力加速度の成分を表す。このことから、私達は加速度計の角度、つまり鳥がどのような姿勢を保っているのか知ることができる（立っているのか、座っているのかなど）。これまでの知見を元に、得られた加速度データを眼で確認することで、幾つかの行動パターンを判別した（Fig. 2）[9,22,23]。対象個体は、計測期間中に2~6回の採餌トリップを行った。個体が深度1m以上に潜った時を潜水と定義した。

Continuous wavelet transformation

私達は、加速度データの振動が非定常状態にあることを考慮し、解析には連続ウェーブレット変換を適用した。ウェーブレット変換は、「異なる場所にそれぞれ焦点を合わせ、観察することを可能にする“数学的顕微鏡”」と表現されている[29]。時系列データを時間成分と周期成分に分解することで、ウェーブレット解析はデータ中に存在する周期成分と、その周期が時間と共にどのように変化していくのかを計算することができる[17]。マザーウェーブレットは、時間成分と周期成分の両方で定義され、時間方向の積分がゼロになる小さい波である。離散配列x_nとして記録された信号の連続ウェーブレット変換は、x_nと（周期スケールと時間を変換した）マザーウェーブレット関数ψの畳み込みとして定義される。

$$W(s, n) = \sum_{n'=0}^{N-1} x_{n'} \psi\left[\frac{(n'-n)\delta t}{s}\right] \quad (1)$$

ここで、s はスケール、nは解析対象となる時刻インデックス、δt は配列の時間間隔、N は全体のデータ数を表す[17]。スケールsと解析対象となる時刻インデックスnが変動することによって、各スケールにおける信号強度とその強度が時間に伴ってどのように変化していくのかを描き出すことができる。

私達は、Morletマザーウェーブレット関数を用いて、異なるスケールsにおける振幅の分布の時間的変化を解析した。Morletウェーブレット関数は以下に定義される。

$$\psi(\eta) = \pi^{-1/4} e^{i\omega_0 \eta} e^{-\eta^2/2} \quad (2)$$

ここで、ω_0は無次元周波数である。Morletウェーブレット関数は三角関数を基礎としている（Fig. 3A）。そのため、時系列データを複数のサインカーブに分解することができ（Fig. 3B）、生態学的現象の解析に広く用いられている[18-20]。無次元周波数ω_0は時系列データを時間成分と周期成分に分解するときの、相互の分解精度のバランスを決定する変数である。ω_0が大きい時、周期成分の分解能は高くなるが、時間成分の分解能は低くなる。ヨーロッパヒメウの加速度データの場合、$\omega_0 = 10$の時に周期成分と時間成分の両方を最もバランスよく分解することが出来たため、この値を解析に用いた。この値の時のマザーウェーブレットは、ほぼ7つの波から構成されている（Fig. 3A）。このマザーウェーブレットを用いて信号の畳み込みを行った結果、入力信号が大体7つくらいの波を構成している時に、時間成分と周期成分の両方の分解能が最も高くなった。

ウェーブレット変換の値 W(*s*, *n*) と入力信号の振幅値を関係付けるため、以下の式を定義した。

$$\mathrm{A}(s,n)=\frac{\sqrt{\left|\mathrm{W}(s,n)\right|^{2}}\,\delta t Ce}{s} \tag{3}$$

ここで *Ce*（1.06392）は、経験的に付加した係数である。この係数 *Ce* を付加することによって、あるスケール *s* と時刻インデックス *n* における、x_n の振幅値が A(*s*, *n*) 値とほぼ等しくなった（Fig. 3B）。

入力信号の周期λとスケール *s* は以下の関係にある[17]。

$$\lambda=\frac{4\pi}{\omega_0+\sqrt{2+\omega_0^2}}s \tag{4}$$

従って、(3)式は、以下の式になる。

$$\mathrm{A}(\lambda,n)=\frac{\sqrt{\left|\mathrm{W}(k\lambda,n)\right|^{2}}\,\delta t Ce}{k\lambda} \tag{5}$$

かつ

$$k=\frac{\omega_0+\sqrt{2+\omega_0^2}}{4\pi} \tag{6}$$

これによって、入力信号がある時刻インデックス *n* において周期λの信号をもつ時、その三角関数成分の振幅値と A(λ, *n*)値は一致する。私達は、その後のクラスター分析を行うことが可能な量にデータ量を減らすため、各周期λにおける A(λ, *n*)の値を 1 秒ごとに平均した値を求めた。これを、ここでは「行動スペクトル」（behavior spectrum）と呼ぶ（Fig. 3B, C, D）。行動スペクトルは、

毎秒ごとの各時刻において、入力信号にそれぞれの周期成分がどのように分布しているかを表している。私達は、0.05 から 1 秒周期の範囲で 64 段階にわたって行動スペクトルの値を計算した。

$$\lambda = C_{\min} \times 2^{\frac{i \log_2 (C_{\max} / C_{\min})}{T}} \quad (i=1,\ 2,\ 3,\ \ldots,\ 64) \qquad (7)$$

ここで、T は時間段階の数（64 段階）、C_{min} は解析を行う最短周期（0.05 秒）、C_{max} は最長周期を（1 秒）を表す。

以上の手順によって、一秒ごとの動物の動きが行動スペクトルによって表現された。各時刻における行動スペクトルの形は入力された信号が持つ周期と振幅の特徴が反映された。通常、動物の動きから生み出される信号は、単一の三角関数の形ではなく、複数のサインカーブが合成された形をしている。それぞれのサインカーブは、異なる振幅を持つと共に整数倍ずつ異なる周波数を有している。この中で、最も低い周波数を fundamental 周波数と呼び、それ以外の物を harmonics と呼ぶ（Fig. 3B）。入力信号中の fundamental 周波数が、動物の動きの周期を表しているとみなした[30]。

本方法で計算された振幅は、動物の動きの大きさと直接に比例しているわけではない。しかし、Morlet ウェーブレット関数を用いた畳み込みにより、加速度信号は複数のサインカーブに分解され、逆にいえば、分解されたサインカーブを合計したものは、（変換に用いた周期の範囲内では）元の入力信号と同じ形になる。そのため、信号を分解することで生成されたサインカーブの組み合わせが類似している時には、それらの信号は類似した動きを示唆していると考えることができるであろう[30]。類似した特徴を示す行動スペクトルどうし

を比べるのであれば、それらのスペクトルが意味する同じ行動パターンの範囲内で個々の動きの相対的な大きさを評価することが出来ると考えられる。

「cone of influence」は、ウェーブレット変換を行った際に、時系列データの始まりと終わりの部分で過小に信号強度が見積もられてしまう現象のことである[17]。cone of influence の影響を避けるために、私達は行動スペクトルの最初と最後を解析の最長周期の 3 倍分の長さだけ切り取り、その後の解析には用いなかった。

Clustering

私達は、エソグラムを作るために *k*-means 法によって、毎秒ごとの行動スペクトルをグループ分けした。*k*-means 法は教師なし分類法であり、各クラスター内で個々の点と各クラスターの重心間のユークリッド距離の二乗を最小にするアルゴリズムである。このアルゴリズムは以下の手順で構成される[21]。

1, クラスター分けする点が存在する空間に *k* 個の点をランダムに配置する。これらの点は、最初の段階の重心である。

2, 個々の点と各重心の間の距離を求め、最も近い距離に位置する重心に対応するクラスターに個々の点を割り当てる。

3, 全ての点を、それぞれのクラスターに割り当てた後、再度 *k* 個のクラスターの重心を計算する。

4, 重心が移動しなくなるまで、手順 2 と 3 を繰り返す。これによって、各点と重心間の距離が最小となる、複数のクラスターを生成することができる。

毎秒ごとの行動スペクトルは 64 段階の周期によって構成された。言い換えれば、各スペクトルは 64 個の値で定義されたといえる。従って、これは 64 次元空間における点の定義と数学的に等しい。私達は、64 次元空間上に存在する点をクラスター分けするのと同じ方法で行動スペクトルのグループ分けを行った[31]。

Acceleration ethogram

エソグラムを作るにあたって、私達はオス、メス 3 羽ずつのヨーロッパヒメウから得られた、1 個体あたり 24 時間分のデータを用いた。まず、surge 方向の加速度成分から行動スペクトルを計算した。繁殖期のヨーロッパヒメウの全ての行動パターンを網羅するには、行動を表す要素（行動要素、behavior element）の数として 20 種類を想定するのが適切であろう、と私達は考えた。そこで、6 羽から得られた行動スペクトルを連結し、ここから *k*-means 法によって、64 次元空間における 20 個の重心を決定した。これらの重心は、それぞれの行動要素における典型的なスペクトルを表現するものとなった。私達は、この 20 個のスペクトル一式を「加速度エソグラム（acceleration ethogram）」とみなした。

Behavior discrimination by acceleration ethogram

次に、16 羽から得られた surge 方向の加速度成分を行動スペクトルに変換した。1 秒ごとに得られたスペクトルは、64 次元空間内の点として捉える事が出来る。これらの点と、加速度エソグラムとしてすでに得られている 20 個の要素に対応する重心とのユークリッド距離の二乗を計算した。各点について、最も小さい値が算出された重心を決定し、それぞれの点を対応する加速度エソグラム中の行動要素に割り当てた。

Behavior discrimination by water depth

我々の方法の正確さを評価するため、16 羽から得られた時系列深度データを用いて各個体が各種の行動に費やした時間配分を算出した。ヨーロッパヒメウの典型的な採餌トリップは、以下のように行われる。ヒメウは繁殖地から採餌場所へ飛行によって移動し、採餌場所では連続して潜水を行う。各潜水の前後は海面上で過ごす。その後、再び繁殖地へ飛行して戻る[32]。時系列深度データから、次の 6 種類の行動パターンを定義した（陸上での行動、移動、潜行、海底での行動、浮上、海面での休息）。潜水中における個々の行動パターンは深度データの変化率を元に定義した（潜行：-0.6 $m s^{-1}$ 以下、海底での行動：-0.3 ~ +0.3 $m s^{-1}$、浮上：1 $m s^{-1}$ 以上）。ヒメウの潜水は、通常、バウト構造をしている。Watanuki et al. [33]に従って、個別のバウトを判別するためのバウト終了基準は 340 秒とした[34]。潜水バウト内における潜水前後の時間は、海面で休息していると定義した。採餌トリップ中の採餌場所との往復に要する飛行時間は 30

分以下であると報告されている[32]。そのため、潜水バウト前後の 30 分間は「移動」行動を行っていると定義した。残りの時間は、陸上ですごしている時間と定義した。時系列深度データから定義された個々の行動パターンに費やした期間に、加速度エソグラムで定義された各行動要素がどれだけの割合で占めているのかを計算した。

Computer application

本研究で示す方法を簡便に行うために、私達は Ethographer という操作が簡単なコンピューターソフトウェアを開発した。プログラムコードは、Igor Pro ver.5(WaveMetrics Inc., Lake Oswego, OR, USA)を用いて、Igor Pro 言語で書かれた。このソフトは、Igor Pro 上で動作する。また、グラフィカルインターフェイスを有していて、簡単に使用方法を習得することができる。Ethographer は、学術目的の場合は無償で使用することができる。(http://bre.soc.i.kyoto-u.ac.jp/bls/index.php?Ethographer).

Results

surge 方向の加速度成分を、加速度エソグラムに従って 20 種類の行動要素に分類した。加速度エソグラムの行動要素は、加速度データの特徴を反映したスペクトルによって記述された（Fig. 4）。幾つかの要素のスペクトルの形は類似していた（例えば、要素 0, 1, 2 や要素 14, 15, 16, 17）（Fig. 4）。これらの

要素は異なる周期と振幅を持つが同じ行動パターンを表していると考えられた。エソグラム中の卓越周期における振幅の範囲は、0.024（要素 7 における 0.11 秒周期）から 5.072（要素 3 における 0.13 秒周期）であった。一般的に、大きな振幅を持つ行動要素ではスペクトルに明瞭なピークが現れたが、小さい振幅だけを有する行動要素では比較的平らなスペクトルになる傾向が認められた。これは、大きな動きを伴う行動パターンは周期的な動きによって構成されているのに対し、そうではない行動パターンには周期性があまり見られないことを意味していると考えられた。

私達の方法を適用した例を Figure 5 に示す。行動スペクトルを見ると、左側の領域では非常に弱い信号だけが認められる（Fig. 5D）。これは、surge 方向の加速度成分がほとんど存在しなかったこと（Fig. 5C）を意味しており、対象個体に動きがなかったことを示唆している。これとは対照的に中央の領域では約 0.18 秒周期の明瞭なピークが認められた。これは、ヒメウの羽ばたき周波数と一致している（5.5Hz, [23]）。右側の領域では、弱く不明瞭な信号がスペクトル中に認められ、0.3 秒以上の弱い周期性を持つ信号の存在を示している。これは、例えば海面での休息行動など、対象個体が不安定な姿勢を取っていることに由来していると考えられる。下のパネルに、*k*-means 法によるグループ分けの結果を示す（Fig. 5E）。明らかに左（陸上、要素 6 と 7）、中央（羽ばたき、要素 1）、右（海面での休息、要素 9）の 3 つの領域に行動が分類されたことが分かる。行動が明らかに 4 つに分類された、もう一つの例を Fig. 6 に示す。それぞれが海面での休息、潜行、海底での行動、浮上に対応していた（Fig. 6D）。

潜行時には、深度に応じて卓越周期が変わり、水面近くでは0.2秒周期の動きが深度 40m では 0.5 秒周期まで長くなっていた。この結果は、ヒメウが潜行するときに、深度に応じて足漕ぎ周波数を5Hz（0.2秒周期）から2Hz（0.5秒周期）まで変化させているという報告と一致した[22]。海底での行動スペクトルの特徴は、0.4 秒と 0.2 秒周期に認められる乱雑で不明瞭なピークに認められた。浮上時には、スペクトル中にピークは認められなかった。海面での休息（要素 9）、海底での行動（主に要素10）、浮上（要素7）の3種類の行動を、*k*-means法によって明瞭に分類することが出来た。グループ分けの結果において興味深い点は、潜行行動の分類結果である。潜行は複数の行動要素の連なりとして分類された（順番に要素13, 14, 15, 16, 17, 10）。これらの行動要素を記述するスペクトルを見てみると、要素13は大きな振幅を持つ複数のピークが存在するという特徴を持っていた（Fig. 4D）。要素14-17のスペクトルは類似していたが、14から17に向かって徐々に卓越周期が長くなり、振幅は小さくなっていった。要素10 は最も長い卓越周期をもち、最も小さい振幅を示した。これらの行動要素の連なりは、潜行時に足漕ぎ行動の周期と強さが深度に伴って変化するという過去の報告と一致するものであった[22]。1回の採餌トリップ全体を俯瞰してみると（Fig. 7）、ほぼ1.8秒周期の動きを表す要素0, 1, 2が潜水バウトの前後に見られ、これが連続的な飛行を示していると考えられた。潜水バウト中に見られる要素14, 15, 16, 17は潜行行動を意味しているのであろう。

加速度エソグラムで規定された各行動要素がどの程度出現するか調べると、深度データによって定義された各行動パターンが見られた期間ごとに、

それぞれの行動要素が占めた割合が異なっていた（Fig. 8）。幾つかの行動パターンでは、1 つか 2 つの行動要素によって、その多くを占められていた。例をあげると、陸上行動（要素 6 と 7）、海面での休息（要素 6 と 9）、海底での行動（要素 10 と 11）、浮上（要素 7）など。面白いことに、海底での行動において要素 10 と 11 が出現する割合は、個体間で大きくばらついていた（Fig. 8）。要素 10 は周期性のない動き、要素 11 は 0.3 秒周期の動きを示唆している（Fig. 4）。メイ島のヨーロッパヒメウは底棲生物を捕食するが、岩場と砂場という二つの環境の異なる採餌場所を利用している[33]。この二種類の採餌場所で、ヒメウの採餌行動は顕著に異なる。岩場では主として海底に沿って水平方向の移動を行うのに対して、砂場では海底の特定の場所に留まり採餌を行う。現段階では、要素 10 と 11 がどのような動作なのかを正確に同定することはできないが、採餌場所利用の個体差によって、この二つの行動要素の出現割合の大きな個体差が生み出されているのかもしれない。潜行の行動パターンでは、足漕ぎの周期と強さに対応する複数の行動要素が現れた（Fig. 6 と Fig. 8D）。移動時（Fig. 8C）には、最も多くの行動要素が出現した（要素 0, 1, 6, 7, 8, 9）。これは、この行動パターンを潜水バウトの前後 30 分間と定義したため、移動のための羽ばたきだけでなく、陸上や海面で過した期間の行動も含まれてしまったことが原因であると考えられる。移動時における要素 7 と 9 の成分は、それぞれ陸上と海面での行動から派生したのではないかと思われる（Fig. 7 を見よ）。移動時に特有の行動要素は、要素 0 と 1 であった。これらの要素の卓越周期は 0.18 秒であり、これはヨーロッパヒメウの羽ばたき周期と一致した。

海面で対象個体が休息している時に得られる加速度信号は、海の状況など周辺環境の影響を受ける可能性がある。私達が解析を行ったデータからは、そのような影響は何ら認められなかったが、周辺環境の違いによって異なる行動スペクトルが計算され、その結果、「海面での休息行動」が加速度エソグラム中の複数の異なる行動要素として認識されてしまう可能性がある。

Discussion

加速度エソグラムを作成するために私達の方法を使用する場合には、個々の動物種がもつ特徴に合わせて、以下の４つの変数を設定する必要がある。一つ目は、体の加速度信号を連続ウェーブレット変換する際の計算に適用する周期の範囲である。私達は、ヨーロッパヒメウを解析するにあたって、0.05~1 秒周期を選んだ。この範囲は、主にヒメウが生み出す動的加速度の周期に対応している[22]。二つ目の変数は、*k*-means 法で作り出す行動要素の数である。私達が *k*-means 法によって加速度エソグラムを作った際には、行動要素の数を 20 まで増やした時に、加速度エソグラムのスペクトルの形はほとんど変化しなくなり、その代り、一つのスペクトルから形の類似した二つのスペクトルが生成されるようになった。そのため、行動要素の数を 20 個と定めれば、全ての行動パターンを網羅できるであろうと私達は考えた。また、数学的に定義された手順によって、*K*-means 法によるグループ分けは行われるため、時としてグループ分けされた結果は、研究者が心に描くものとは違ってしまう場合もある。この

問題の現実的な解決策としては、まず行動要素の数として十分に大きな数を設定し、その後、研究者が同じ行動を表していると考える行動要素を合体させると良いであろう。三つ目の変数は、Morlet マザーウェーブレット関数のω_0の値である。連続ウェーブレット変換の性質上、周期的な動きの方が周期的でない動きに比べて検出しやすい。ω_0の値を変更することで、時間成分と周期成分のどちらの検出精度を高くして動きを検出するかを調節することができる。四つ目の変数は、単一の行動スペクトルの時間の長さである。私達は、A(λ, *n*)の値を 1 秒ごとに平均して行動スペクトルを計算した。一般的な目的でヨーロッパヒメウの各行動を調べる場合であれば、1 秒間は十分に高い時間精度だと考えたためである。この時間の長さは、一つの行動単位に対して、それぞれの動物種が取りうる最小時間を考慮した上で調節するべきであろう。私達のコンピューターソフトウェア「Ethographer」ではこれらの変数を調節することができる。

k-means 法によるグループ分けでは、それぞれの行動要素で規定される行動は、定常的な周期と振幅を持つことを仮定している。そのため、例えば、「歩く」と「走る」を正確に区別することは難しい。なぜなら、この二つの行動は緩やかに連続した行動で、明確な境界線を定めることが難しいからである。グループ分けのアルゴリズムは、「歩く」または「走る」の行為に対して、微妙に異なる周期を持つ複数の行動要素を出力することだろう（Fig. 6D の潜行の部分を見よ）。このような種類の行動の場合は、その正確な定義は解析のたびに変動してしまうかもしれない。

今回の解析方法には、コンピューターの性能に起因する 2,3 の制約が存在することが判明している。最も重要なものは、*k*-means 法でグループ分けすることができるデータの数についてである。本研究では、16 羽のヒメウのうちから 6 羽を選び、各個体で 24 時間ずつ記録されたデータ（合計 24 x 6 = 144 時間）を用いて、グループ分けを行った。つまり、加速度エソグラムを作るのに用いた行動スペクトルの数は約 500,000 個であった。私達が解析を行ったコンピューター（Intel Core 2 Duo 6600 CPU, 2 GB RAM）では、これが加速度エソグラム生成を行うことが可能な最大容量であった。もう一つの制約は、*k*-means 法自体に関係することである。*k*-means 法によるグループ分けの出力結果は解析を行うたびに、（ほとんど同じではあるものの）全く同じ結果を常に出力するとは限らない。なぜならば、*k*-means 法は最適解を求める方法ではなく、最適解に近い値を求める方法だからである。現状における計算量の問題を考慮した場合、最適解を求めることは現実な方法ではないと私達は考えた（例えば、階層的クラスタリング）。

本研究の結果では、体の加速度を元にグループ分けした行動は、時系列深度データから定義した行動パターンと概ね一致した。しかし、本方法では陸上行動と浮上を区別することはできなかった。私達は行動スペクトルを算出するために、体の surge 方向の加速度成分のうち、主に動的加速度の情報を用いた。そのため、動物の体に動きがない時には、たとえ姿勢が異なっていたとしても、行動スペクトルは同じになってしまった。本研究で作られた加速度エソグラムは、対象個体の surge 方向の加速度成分の情報だけから作られた行動のカタログ

である。完全なエソグラムを作成するためには、深度ロガーや GPS ロガーなどと共に、体の加速度の長周期成分から得られる姿勢の情報が必要かもしれない。

今日に至るまで、追跡することが困難な野生動物の行動を定量化することは非常に困難である。保全生物学の観点で言えば、絶滅危惧種の採餌生態に関する情報が欠如していると、有効な保全戦略の策定の障害になると考えられる。このような動物種の、これまで知られていない生態の一面を解明するのに、私達の方法は役に立つかもしれない。特筆すべき点は、私達の方法は教師なし分類法を用いていることである。そのため、研究者がこれまで直接観察したことが無い新規の行動パターンであったとしても、本方法を使えば抽出できる可能性がある。本論文で示した方法は、他の動物種においても適切なエソグラムを生成できることを保証するものではない。しかし、観察することが困難な場所に生息しているために、これまで研究されてこなかった動物種の行動を調べる場合に、本方法は大きな力を発揮すると思われる。

Acknowledgments

We thank G. T. Crossin for comments on the manuscript and two anonymous referees for reviewing the manuscript and helpful suggestions.

References

1. Martin P, Bateson P (2007) Measuring Behavior: An Introductory Guide, Third Edition. Cambridge: Cambridge University Press. 187 p.
2. Cooke SJ, Hinch SG, Wikelski M, Andrews RD, Kuchel LJ, et al. (2004) Biotelemetry: a mechanistic approach to ecology. Trends Ecol Evol 19: 334–343.
3. Boyd IL, Kato A, Ropert-Coudert Y (2004) Bio-logging science: sensing beyond the boundaries. Mem Nat Inst Pol Res Tokyo 58: 1-14.
4. Hooker SK, Biuw M, McConnell B, Miller PJO, Sparling CE (2007) Bio-logging science: Logging and relaying physical and biological data using animal-attached tags. Deep-Sea Res II 54: 177-182.
5. Rutz C, Hays GC (2009) New frontiers in biologging science. Biol Lett. Published online (doi:10.1098/rsbl.2009.0089).
6. Tremblay Y, Cherel Y, Oremus M, Tveraa T, Chastel O (2003) Unconventional ventral attachment of time-depth recorders as a new method for investigating time budget and diving behaviour of seabirds. J Exp Biol 206: 1929-1940.
7. Takahashi A, Sato K, Naito Y, Dunn MJ, Trathan PN, at al. (2004) Penguin-mounted cameras glimpse underwater group behaviour. Proc R Soc B 271: S281–S282.
8. Rutz C, Bluff LA, Weir AAS, Kacelnik A (2007) Video cameras on wild birds.

Science 318: 765.

9. Yoda K, Naito Y, Sato K, Takahashi A, Nishikawa J, et al. (2001) A new technique for monitoring the behaviour of free-ranging Adélie penguins. J Exp Biol 204: 685-690.

10. Ropert-Coudert Y, Grémillet D, Kato A, Ryan PG, Naito Y, at al. (2004) A fine-scale time budget of Cape gannets provides insights into the foraging strategies of coastal seabirds. Anim Behav 67: 985-992.

11. Watanabe S, Izawa M, Kato A, Ropert-Coudert Y, Naito Y (2005) A new technique for monitoring the detailed behaviour of terrestrial animals: a case study with the domestic cat. Appl Anim Behav Sci 94: 117-131.

12. Wilson RP, Shepard ELC, Liebsch N (2008) Prying into the intimate details of animal lives: use of a daily diary on animals. Endang Species Res 4: 123-137.

13. Laich AG, Wilson RP, Quintana F, Shepard ELC (2008) Identification of imperial cormorant *Phalacrocorax atriceps* behavior using accelerometer. Endang Species Res. Published online (doi:10.3354/esr00091).

14. Lagarde F, Guillon M, Dubroca L, Bonnet X, Ben Kaddour K, et al. (2008) Slowness and acceleration: a new method to quantify the activity budget of chelonians. Anim Behav 75: 319-329.

15. Guilford T, Meade J, Willis J, Phillips RA, Boyle D, et al. (2009) Migration and stopover in a small pelagic seabird, the Manx shearwater *Puffinus puffinus*: insights from machine learning. Proc R Soc B 276:1215-1223.

16. Percival DB (1995) On the estimation of the wavelet variance. Biometrika 82: 619-631.
17. Torrence C, Compo GP (1998) A practical guide to wavelet analysis. Bull Am Meteor Soc 79: 61-78.
18. Grenfell BT, Bjørnstad ON, Kappey J (2001) Travelling waves and spatial hierarchies in measles epidemics. Nature 414: 716-723.
19. Jenouvrier S, Weimerskirch H, Barbraud C, Park Y-H, Cazelles B (2005) Evidence of a shift in the cyclicity of Antarctic seabird dynamics linked to climate. Proc R Soc B 272: 887-895.
20. Cazelles B, Chavez M, Berteaux D, Ménard F, Vik JO, et al. (2008) Wavelet analysis of ecological time series. Oecologia 156: 287-304.
21. Jain AK, Murty MN, Flynn PJ (1999) Data clustering: a review. ACM Comput Surv 31: 264-323.
22. Watanuki Y, Takahashi A, Daunt F, Wanless S, Harris M, et al. (2005) Regulation of stroke and glide in a foot-propelled avian divers. J Exp Biol 208: 2207-2216.
23. Sato K, Daunt F, Watanuki Y, Takahashi A, Wanless S (2008) A new method to quantify prey acquisition in diving seabirds using wing stroke frequency. J Exp Biol 211: 58-65.
24. Wanless S, Harris MP (1992) At-sea activity budgets of a pursuit-diving seabirds monitored by radio telemetry. In: Priede IG, Swift SM, editors. Wildlife telemetry: remote monitoring and tracking animals. London: Ellis Horwood. pp. 591-598.

25. Wanless S, Burger AE, Harris MP (1991) Diving depths of shags *Phalacrocorax aristotelis* on the Isle if May. Ibis 133: 37-42.

26. Wanless S, Corfield T, Harris MP, Buckland ST, Morris JA (1993) Diving behaviour of the shag *Phalacrocorax aristotelis* (Aves: Pelecaniformes) in relation to water depth and prey size. J Zool Lond 231: 11-25.

27. Wanless S, Harris MP, Russell AF (1993) Factors influencing food-load size brought in by shags *Phalacrocorax aristotelis* during chick rearing. Ibis 135: 19-24.

28. Watanuki Y, Takahashi A, Daunt F, Sato K, Miyazaki N, et al. (2007) Underwater images from bird-borne cameras provide clue to poor breeding success of European shags in 2005. British Birds 100: 466-470.

29. Karlsson S, Yu J, Akay M (2000) Time-frequency analysis of myoelectric signals during dynamic contractions: a comparative study. IEEE Trans Biomed Eng 47: 288-237.

30. Young SS (2001) Computerized data acquisition and analysis for the life sciences. Cambridge: Cambridge University Press. 237 p.

31. Tavazoie S, Hughes JD, Campbell MJ, Cho RJ, Church GM (1999) Systematic determination of genetic network architecture. Nat Genet 22: 281-285.

32. Daunt F, Wanless S, Harris MP, Money L, Monaghan P (2007) Older and wiser: improvements in breeding success are linked to better foraging performance in European shags. Funct Ecol 21: 561-567.

33. Watanuki Y, Daunt F, Takahashi A, Newell M, Wanless S, et al. (2008) Microhabitat use and prey capture of a bottom-feeding top predator, the European shag, shown by camera loggers. Mar Ecol Prog Ser 356: 283-293.

34. Gentry RL, Kooyman GL (1986) Methods of dive analysis. In: Gentry RL, Kooyman GL, editors. Fur Seals. Princeton: Princeton University Press. pp 28-40.

Figure Legends

Figure 1.

データロガーを装着した位置と体の長軸（surge）方向の向き。

Figure 2.

ヨーロッパヒメウから得られたSurge方向の加速度成分と深度データ。図はそれぞれ、(A)陸上での行動、(B)海面での休息、(C)羽ばたき、(D)潜行、(E)海底での行動、(F)浮上を表す。

Figure 3.

連続ウェーブレット変換の概略図。(A) ω_0 = 10の時のMorletマザーウェーブレット関数。実線と波線はそれぞれ実数部と虚数部を表す。(B)行動スペクトルの例。

モデルデータは0.5秒周期のコサインカーブ(time <= 00:00:07)とそれぞれが0.25秒と0.5秒の周期をもつ二つのコサインカーブの合成波(time > 00:00:07)である（上）。個々のコサインカーブの振幅は1である。モデルデータから生成された行動スペクトル（下）。変換された周期の範囲は0.1秒から1秒である。(C)時刻0:00:03と(D) 時刻0:00:11における行動スペクトル。行動スペクトルでピークを示した周期における振幅の値は、変換前のモデルデータに含まれる信号の振幅の値と一致する。

Figure 4.

加速度エソグラムに含まれる20種類の行動要素。エソグラムは6羽のヒメウ（オス3羽とメス3羽）から24時間ずつ得られたsurge方向の加速度成分から作られた。縦軸は加速度の振幅を表す。横軸は加速度の周期を表す。見やすくするために、エソグラムは6つに分割して、図示してある。

Figure 5.

エソグラムを生成する工程の見取り図（飛行中のデータ）。(A)各行動パターン（陸上行動、羽ばたき、海面での休息）はデータを眼で確認して判別した。(B)深度データ。(C)surge方向の加速度成分。(D)行動スペクトル。(E)加速度エソグラムで規定された行動要素。眼で確認することで判別された行動パターンと自動生成された行動要素が比較できる。

Figure 6.

エソグラムを生成する工程の見取り図（潜水中のデータ）。(A)各行動パターン（海面での休息、潜行、海底での行動、浮上）はデータを眼で確認して判別した。(B)深度データ。(C)surge方向の加速度成分。(D)行動スペクトル。(E)加速度エソグラムで規定された行動要素。眼で確認することで判別された行動パターンと自動生成された行動要素が比較できる。

Figure 7.

加速度エソグラムと深度データから定義された行動パターンとの間に見られる一致。(A)深度データ。(B)深度データによって定義された行動パターン。海面での休息、潜行、海底での行動、浮上は、まとめてDive Boutとして示した。(C)加速度エソグラムで規定された各行動要素が毎分ごとに占めた割合

Figure 8.

それぞれの行動パターン中に各行動要素が占めた割合。それぞれの行動パターンの期間中に、加速度エソグラムで規定された各行動要素が占めた割合を箱ひげ図で表した（n=16）。それぞれの行動パターンは深度データによって定義された。それぞれの図は、(A)陸上での行動、(B)海面での休息、(C)移動、(D)潜行、(E)海底での行動、(F)浮上を行っている期間に各行動要素が占めた割合を表している。箱ひげ図は、中央値（中心線）、上位と下位四分位（箱の境界線）、10%と90%パーセンテイル（ひげの先端）をそれぞれ示している。
